# The relationship between the BMI‐adjusted weight loss grading system and quality of life in patients with incurable cancer

**DOI:** 10.1002/jcsm.12499

**Published:** 2019-11-06

**Authors:** Louise Daly, Ross Dolan, Derek Power, Éadaoin Ní Bhuachalla, Wei Sim, Marie Fallon, Samantha Cushen, Claribel Simmons, Donald C. McMillan, Barry J. Laird, Aoife Ryan

**Affiliations:** ^1^ School of Food and Nutritional Sciences, College of Science, Engineering and Food Science University College Cork Cork Ireland; ^2^ Academic Unit of Surgery University of Glasgow Glasgow UK; ^3^ Department of Medical Oncology Mercy and Cork University Hospital Cork Ireland; ^4^ Cork Cancer Research Centre University College Cork Cork Ireland; ^5^ Edinburgh Cancer Research Centre Institute of Genetics and Molecular Medicine University of Edinburgh Edinburgh UK

**Keywords:** Cancer, Malnutrition, Weight loss, Cachexia, Quality of life, Survival

## Abstract

**Background:**

Weight loss (WL) has long been recognized as an important factor associated with reduced quality of life (QoL) and reduced survival in patients with cancer. The body mass index (BMI)‐adjusted weight loss grading system (WLGS) has been shown to be associated with reduced survival. However, its impact on QoL has not been established. The aim of this study was to assess the relationship between this WLGS and QoL in patients with advanced cancer.

**Methods:**

A biobank analysis was undertaken of adult patients with advanced cancer. Data collected included patient demographics, Eastern Cooperative Oncology Group performance status, and anthropometric parameters (BMI and %WL). Patients were categorized according to the BMI‐adjusted WLGS into one of five distinct WL grades (grades 0–4). QoL was collected using the European Organization for Research and Treatment of Cancer Quality of Life Questionnaire‐C30. The Kruskal–Wallis test and multivariate logistic regression analyses were used to assess the relationship between the WLGS and QoL scores. Overall survival was assessed using Kaplan–Meier curve and Cox proportional hazard models.

**Results:**

A total of 1027 patients were assessed (51% male, median age: 66 years). Gastrointestinal cancer was most prevalent (40%), and 87% of patients had metastatic disease. Half (58%) of patients had a WL grade of 0–1, while 12%, 20%, and 10% had WL grades of 2, 3, and 4, respectively. Increasing WL grades were significantly associated with poorer QoL functioning and symptoms scales (all *P* < 0.05). Physical, role, and emotional functioning decreased by a median of >20 points between WL grade 0 and WL grade 4, while appetite loss, pain, dyspnoea, and fatigue increased by a median score >20 points, indicative of a large clinical significant difference. Increasing WL grades were associated with deteriorating QoL summary score. WL grades 2, 3, and 4 were independently associated with a QoL summary score below the median (<77.7) [odds ratio (OR) 1.69, *P* = 0.034; OR 2.06, *P* = 0.001; OR 4.29, *P* < 0.001, respectively]. WL grades 3 and 4 were independently associated with reduced overall survival [hazard ratio 1.54 (95% confidence interval: 1.22–1.93), *P <* 0.001 and hazard ratio 1.87 (95% confidence interval: 1.42–2.45), *P* < 0.001, respectively].

**Conclusions:**

Our findings support that the WLGS is useful in identifying patients at risk of poor QoL that deteriorates with increasing WL grades. WL grade 4 is independently associated with a particularly worse prognosis and increased symptom burden. Identification and early referral to palliative care services may benefit these patients.

## Introduction

Weight loss (WL) has long been recognized as an important and prognostic clinical feature in patients with cancer. Involuntary WL is estimated to affect between 30% and 70% of patients and has been associated with reduced quality of life (QoL) and physical function, poorer tolerance to anti‐cancer therapy, and shortened survival in patients with cancer.^1^, ^2^, ^3^, ^4^ WL is a cardinal feature of cancer cachexia, a condition characterized by the loss of muscle with or without the loss of fat mass, leading to progressive functional impairment.^5^ However, current definitions and thresholds for defining clinically important WL are unclear, particularly in the face of a global obesity epidemic. The use of minimum reported degrees of WL (e.g. >5% or 10% over 3–6 months) is arbitrary and heterogenous and not based on specific values that relate to adverse clinical outcome. It has been suggested that the severity of WL should be evaluated based on the rate of WL in the context of initial body reserves and that thresholds for clinically important WL should relate optimally to meaningful patient‐centred outcomes, such as decreased survival.^5^

In 2015, Martin *et al*. aimed to redefine clinically important WL that was prognostic of outcome in a large dataset of >10 000 cancer patients across Europe and Canada.^6^ This dataset was used to develop and validate a new grading system for cancer‐associated WL based on risk stratification with survival as the outcome. The authors used a 5 × 5 matrix analysis of 25 possible combinations of %WL and body mass index (BMI) and combining groups with similar hazard ratios (HRs). From this, they devised the BMI‐adjusted weight loss grading system (WLGS) that composed of five distinct WL grades with significantly different survival rates. Median survival was longest for WL grade 0 (20.9 months) and shortest for WL grade 4 (4.3 months). Importantly, these observations were independent of tumour site, stage, and performance status (PS).^6^ The WLGS marked a step forward in redefining clinically important WL and has been included in the current international clinical practice guidelines for nutrition and cancer.^7^

Since then, the WLGS has had its prognostic validity confirmed in a cohort of oncology patients.^8^ Further, the WLGS was also associated with cachexia‐related domains such as reduced dietary intake, anorexia, reduced PS, and increased fatigue, suggesting that the WLGS may be useful in cachexia classification.^8^ However, the relationship between the WLGS and QoL is unclear. The relationship between WL in isolation and reduced QoL has long been recognized, and this was confirmed in a systematic review in 2013, whereby a negative relationship between WL and QoL was reported in 23 of the 27 studies.^9^ However, the WLGS that incorporates BMI in addition to WL and is the most robust prognostic framework using these domains published to date may also relate to QoL; however, this has yet to be elucidated.

Therefore, the aim of the present study was to first assess the prognostic validity of the WLGS in an external cohort of patients with advanced cancer and, second, examine the relationship between the WLGS and QoL in patients with advanced cancer and assess if increasing WL grades are capable of identifying patients at risk of impaired QoL.

## Patients and methods

A biobank analysis of patients with cancer was performed. These data were collected prospectively from 18 centres (11 cancer centres and 7 specialist palliative care units) across the UK and Ireland^10^, ^11^ between 2011 and 2016. Individual centres were opened at staggered time points, and a convenience sampling approach was adopted. For the primary data collection, both oncology inpatients and outpatients were recruited. Eligible patients were >18 years of age and have advanced cancer [defined as metastatic cancer (histological, cytological, or radiological evidence), locally advanced or receiving anti‐cancer therapy with palliative intent]. Willing participants provided written informed consent. Exclusion criteria included patients that were under the age of 18 years and those that were unwilling or unable to participate due to cognitive impairment. All participants provided written informed consent, ethical approval was given (UK—12/SS/0181 and EMC 4(g) 2015 Ireland), and the studies were conducted according to good clinical practice and applicable laws.

### Patient information recorded

On assessment, patient's weight, height, and BMI were recorded [weight (kg)/height (m^2^)]. Body weight was measured to within 0.1 kg, using a digital scale. Height was measured to within 0.5 cm using a wall‐mounted stadiometer. Patient‐reported history of WL in the preceding 3 months was assessed and recorded in a study‐specific questionnaire and when possible verified from patient's medical records. From this, percentage WL (%WL) was calculated [weight lost (kg)/current weight (kg) × 100]. WL grade was assessed and given a score of 0–4 by combining WL and current BMI according to Martin *et al*. (*Table* 1).^6^ Clinical and pathological data were collected and included information on patient demographics (age and gender), primary tumour site, stage, and extent of metastatic disease (if present).

**Table 1:** Grade of weight loss (0–4) based on percentage weight loss and current body mass index^6^

Body mass index (kg/m^2^)						
Weight loss (%)	≥28	25–27.9	22–24.9	20–21.9	<20
±2.4	0	0	1	1	3
2.5–5.9	1	2	2	2	3
6–10.9	2	3	3	3	4
11–14.9	3	3	3	4	4
≥15	3	4	4	4	4

Performance status was assessed by the Eastern Cooperative Oncology Group (ECOG) score.^12^ ECOG PS was assigned according to patient‐reported daily physical function: 0 = fully active with no restrictions; 1 = restricted in physically strenuous activity but ambulatory and able to carry out light work; 2 = ambulatory and capable of all self‐care but unable to carry out any work activities; 3 = capable of only limited self‐care; and 4 = completely disabled and totally confined to bed or chair. Patients were followed prospectively until the date of censoring (11/06/2018) or date of death from any cause (if present). Survival time was calculated from the date of recruitment to the date of death or censoring, whichever came first.

Following written informed consent, patients were provided instruction on how to complete the QoL questionnaire, and this was performed during their visit to their treating cancer centre or specialist palliative care unit. QoL was recorded using the European Organization for Research and Treatment of Cancer Quality of Life Questionnaire (EORTC QLQ‐C30 version 3.0), which is a 30‐item cancer‐specific questionnaire including five functional scales (physical, emotional, cognitive, social, and role), three symptom scales (fatigue, pain, and nausea/vomiting), a global health/QoL scale, and six single items (dyspnoea, insomnia, appetite loss, constipation, diarrhoea, and financial impact of disease).^13^ The 28 items measuring functional and symptom scales have a numeric scale: 1 (not at all), 2 (a little), 3 (quite a bit), and 4 (very much). The two items concerning global QoL have a scale of 1 (very poor) to 7 (excellent). The raw scores were linearly transformed to give standard scores in the range of 0–100 for each of the scales and single items as described by the EORTC.^13^ Higher scores for the functional or global QoL scale represent a high level of functioning or QoL, whereas higher scores on the symptom scales represent worse symptomatology. With regard to EORTC QLQ‐C30L, a difference in score of 5–10 was considered a small clinical difference, a difference in score of 10–20 was considered a moderate difference, and a difference in score ≥20 was considered a large clinically significant difference.^14^

### Statistical analysis

Statistical analysis was completed using SPSS (version 24.0, SPSS Inc., Chicago, IL, USA). Data are expressed as median and interquartile range (IQR). Comparisons between groups of patients were assessed using *χ* ^2^ test for categorical variables and unpaired *t*‐tests and Mann–Whitney *U*‐tests to test for differences in continuous variables depending if the data were parametric or non‐parametric, respectively.

The first step in this analysis was to confirm the prognostic utility of the WLGS in this group of patients before embarking on the main analysis, which was to assess the relationship between the WLGS and QoL.

Survival curves were constructed using the Kaplan–Meier technique, and log‐rank test was used to compare survival between groups of patients. Univariate and multivariate analyses of overall survival (OS) were performed using Cox proportional hazard model. HRs with 95% confidence intervals (CIs) were calculated. The predefined adjustment variables in the multivariable models were age, sex, primary disease site, and ECOG PS. To examine the differences between BMI‐adjusted WLGS and median EORTC QoL functional and symptom scores, the non‐parametric Kruskal–Wallis test was used, and Jonckheere–Terpstra test was used to test for a linear trend across BMI‐adjusted WLGS. Summary QoL score, ranging from 0 to 100 (high scores indicating better QoL), was median dichotomized for multivariate logistic regression analyses in order to assess the impact of the BMI‐adjusted WLGS on overall QoL.^15^ Patients with a summary QoL score below the median were given a score of 1, while those with a score above the median were given a score of 0. Thus, odds ratios (ORs) greater than 1.0 indicate a greater likelihood of worse QoL. All *P*‐values were two sided, and the level of significance was *P* < 0.05.

## Results

### Patient characteristics and demographics

A total of 1027 patients were assessed with characteristics presented in *Table* 2. In brief, 51% of patients were male with a median age (IQR) of 66 (57–74) years. Gastrointestinal cancers were most prevalent, accounting for 40% of cases, followed by lung cancer (26%). The majority of patients (81%) had received chemotherapy in the previous 3 months, and 59% had a good PS (ECOG 0–1). Distant metastatic disease was present in 862 (87%) patients, and the most common sites were liver (35%), lung (25%), or bone (19%). Patients were a median of 4.6 months from diagnosis at point of data collection (IQR 3.0–13.0 months).

**Table 2:** Demographic and clinical characteristics of patients included in this study

	*n*	% of patients
Sex		
Male	524	51
Female	503	49
Age (years)		
<65	483	47
65–74	300	29
≥75	244	24
Primary cancer		
Gastrointestinal	411	40
Lung	266	26
Other	350	34
Metastatic disease^a^		
Yes	862	87
No	132	13
Performance status (ECOG)^b^		
0–1	575	59
2	292	30
3	96	10
4	16	1

Percentages given for total available. Other group consists of breast (*n* = 91), gynaecological (*n* = 64), genitourinary (*n* = 69), neurological (*n* = 10), haematological (*n* = 43), melanoma (*n* = 40), unknown primary (*n* = 12), and other (*n* = 21). ECOG, Eastern Cooperative Oncology Group.

a Available in 994.

b Available in 979.

Anthropometric and WL data are presented in *Table* 3. In terms of the BMI‐adjusted WLGS, 58% of patients had a WL grade of 0–1, while 12%, 20%, and 10% had WL grades of 2, 3, and 4, respectively. Patients in the five WL categories did not differ in relation to sex or age; however, high grade WL (≥2) was more common in those with gastrointestinal (45%), lung (46%), gynaecological (48%), and neurological (56%) cancer compared with breast cancer (26%), genitourinary cancer (23%), and melanoma (31%) (*P* = 0.002). The prevalence of patients with high grade WL (grade ≥2) increased with deteriorating ECOG PS [37% (ECOG 0–1), 50% (ECOG 2), 55% (ECOG 3), 82% (ECOG 4), *P* < 0.001].

**Table 3:** Anthropometric and nutritional status characteristics of patients included in this study

Characteristic	Total *n* = 1027
BMI (kg/m^2^)^a^, *n* (%)	
Underweight (<18.5 kg/m^2^)	68 (7)
Healthy weight (18.5–24.9 kg/m^2^)	402 (42)
Overweight (25–29.9 kg/m^2^)	299 (32)
Obese (≥30 kg/m^2^)	180 (19)
Weight loss (>2%)^b^, *n* (%)	399 (42)
Median weight loss (IQR) (%)	−7.3 (−4.4 to −12.6)
Weight stable (±2%), *n* (%)	449 (47)
Weight gain (>2%), *n* (%)	103 (11)
Median weight gain (IQR) (%)	+4.7 (+3.2 to +8.1)
BMI‐adjusted WLGS^c^, *n* (%)	
0	334 (35)
1	214 (23)
2	112 (12)
3	186 (20)
4	97 (10)

Percentages given for total available. BMI, body mass index; BMI‐adjusted WLGS, body mass index‐adjusted weight loss grading system; IQR, interquartile range.

a Available in 949.

b Available in 951.

c Available in 943.

### Weight loss grading system and survival

**Figure 1:**
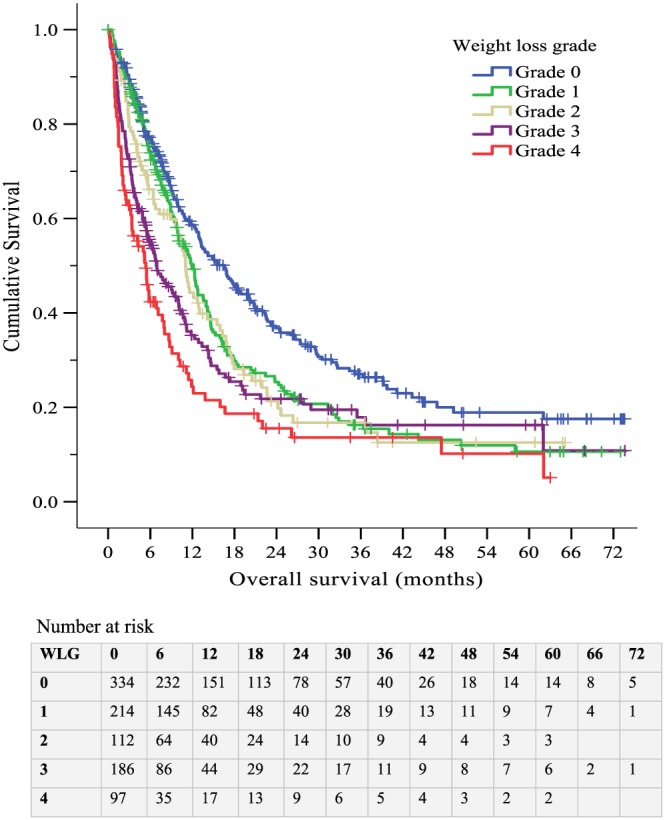
Kaplan–Meier curve displaying cumulative survival by weight loss grade. Censored cases indicated by +.

The median OS for the entire cohort was 10.4 months (95% CI: 9.4–11.4 months). At the time of censoring, 317 of the 1027 patients (31%) were still alive. Median follow‐up time for these patients was 31.8 months (95% CI: 27.9–35.6). Survival worsened with increasing WL grades (*Figure* 1). Median OS decreased from 16.6 months (95% CI: 13.6–19.6) in WL grade 0 to 5.4 months (95% CI: 3.9–6.8) in WL grade 4 (log rank: *P* < 0.001). On multivariate regression analysis, WL grades 3 and 4 remained independently associated with reduced survival [HR 1.54 (95% CI: 1.22–1.93), *P <* 0.001 and HR 1.87 (95% CI: 1.42–2.45), *P* < 0.001, respectively] (*Table* 4).

**Table 4:** Estimated crude and adjusted hazard ratios for Cox proportional hazard model assessing the effect of variables associated with survival

	*n*	Univariate analysis		Multivariate analysis^a^	
		HR (95% CI)	*P*‐value	HR (95% CI)	*P*‐value
Sex					
Men	524	1.00		1.00	
Women	503	0.88 (0.76–1.02)	0.092	0.94 (0.79–1.10)	0.428
Age (years)					
<65	483	1.00		1.00	
64–74	300	1.10 (0.93–1.31)	0.276	1.02 (0.85–1.23)	0.854
≥75	244	1.21 (1.00–1.46)	0.048	0.93 (0.79–1.10)	0.481
Cancer site					
Gastrointestinal	411	1.00		1.00	
Lung	266	1.36 (1.14–1.64)	0.001	1.26 (1.03–1.54)	0.024
Other	350	0.64 (0.54–0.77)	<0.001	0.68 (0.56–0.83)	<0.001
ECOG					
0–1	575	1.00		1.00	
2	292	2.01 (1.69–2.39)	<0.001	1.84 (1.53–2.21)	<0.001
3	96	3.83 (3.00–4.89)	<0.001	3.31 (2.37–4.14)	<0.001
4	16	22.69 (13.47–38.25)	<0.001	15.45 (8.20–29.11)	<0.001
BMI‐adjusted WLGS					
Grade 0	312	1.00		1.00	
Grade 1	201	1.31 (1.07–1.62)	0.010	1.16 (0.93–1.44)	0.189
Grade 2	107	1.44 (1.11–1.86)	0.006	1.27 (0.97–1.65)	0.084
Grade 3	182	1.71 (1.37–2.13)	<0.001	1.54 (1.22–1.93)	<0.001
Grade 4	97	2.28 (1.75–2.97)	<0.001	1.87 (1.42–2.45)	<0.001

Cases available for analysis: *n* = 899. BMI‐adjusted WLGS, body mass index‐adjusted weight loss grading system; CI, confidence interval; ECOG PS, Eastern Cooperative Oncology Group performance status; HR, hazard ratio.

a Multivariate model adjusted for sex, age, cancer site, and ECOG PS.

### Weight loss grading system and quality of life

**Figure 2:**
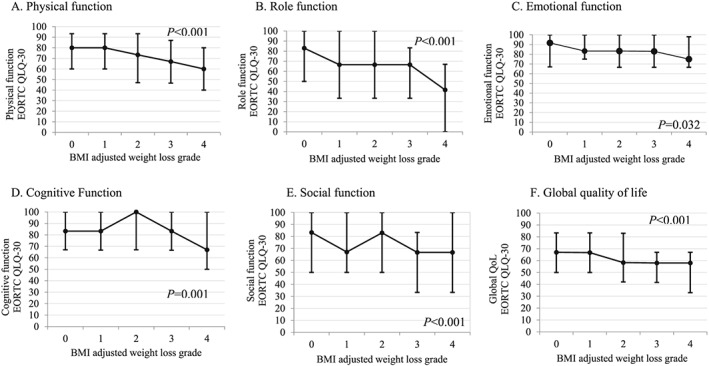
Relationship between body mass index (BMI)‐adjusted weight loss grades (0–4) and median (interquartile range) functional scores from quality of life [European Organization for Research and Treatment of Cancer Quality of Life Questionnaire (EORTC QLQ‐30)] assessed by the Kruskal–Wallis test.

**Figure 3:**
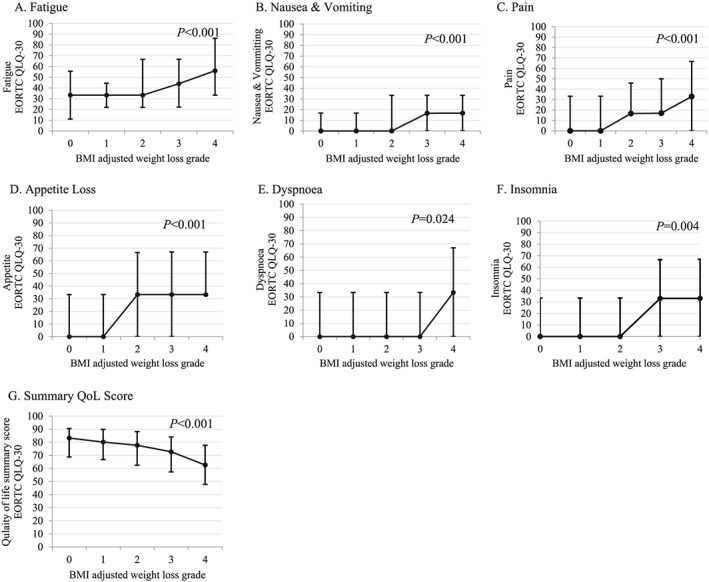
Relationship between body mass index (BMI)‐adjusted weight loss grades and median (interquartile range) symptom scores from quality of life (QoL) domains [European Organization for Research and Treatment of Cancer Quality of Life Questionnaire (EORTC QLQ‐30)] assessed by the Kruskal–Wallis test.

Quality of life data (EORTC QLQ‐C30) were available in 1000 patients. Increasing WL grades were significantly associated with poorer QoL for a number of functional (physical, role, emotional, cognitive, social, and global health) and symptom (fatigue, nausea and vomiting, pain, appetite loss, and dyspnoea) scales and summary QoL score (*Figures* 2A–2F and 3A–3G). The greatest difference in median functional QoL scores between BMI‐adjusted WL grades 0 and 4 was observed in role functioning (83 vs. 41.6, *P* < 0.001) and physical functioning (80 vs. 60, *P* < 0.001). The greatest difference in median QoL symptom scores between BMI‐adjusted WL grades 0 and 4 was observed in appetite loss (0 vs. 33.3, *P* < 0.001), pain (0 vs. 33.3, *P* < 0.001), dyspnoea (0 vs. 33.3, *P* < 0.001), insomnia (0 vs. 33.3, *P* < 0.001), and fatigue (33.3 vs. 56, *P* < 0.001), all consistent with a large clinically meaningful difference (Δ > 20 points). No significant difference in median symptom scores for diarrhoea, constipation, or financial impact score was observed across the BMI‐adjusted WLGS; in fact, for each WL grade, the median score for diarrhoea, constipation, and financial impact was 0. Deteriorations in EORTC QLQ‐C30 functional and symptom scales most commonly occurred in BMI‐adjusted WL grade ≥2; however, in some instances, even WL grade 1 was associated with lower functional (role, emotional, and social function) scores but not symptom scores. Importantly, increasing WL grades were associated with deteriorating QoL summary score across all WL grades (83.2 in WL grade 0 vs. 62.7 in WL grade 4). On multivariate logistic regression, ECOG PS, cancer site, and BMI‐adjusted WL grades were independently associated with a QoL summary score below the median (<77.7) (*Table* 5). Although WL grade 1 was not associated with poorer overall QoL summary score, compared with WL grade 0, WL grade 2 [OR 1.69 (95% CI: 1.04–2.73), *P* = 0.034], WL grade 3 [OR 2.06 (95% CI: 1.37–3.11), *P* = 0.001], and WL grade 4 [OR 4.29 (95% CI: 2.44–7.55), *P* < 0.001] were independently associated with an increased risk of having a QoL summary score below the median.

**Table 5:** Estimated crude and adjusted odds ratios for logistic regression hazard model assessing the effect of weight loss grading system on overall summary quality of life score (below the median <77.7)

	*n*	Univariate analysis		Multivariate analysis^a^	
		OR (95% CI)	*P*‐value	OR (95% CI)	*P*‐value
Sex					
Men	499	1.00			
Women	501	1.35 (1.05–1.73)	0.019	1.16 (0.85–1.57)	0.354
Age (years)					
<65	476	1.00		1.00	
64–74	289	0.95 (0.71–1.27)	0.730	0.79 (0.56–1.12)	0.193
≥75	235	1.00 (0.74–1.38)	0.956	0.59 (0.40–0.88)	0.009
Cancer site					
Gastrointestinal	400	1.00		1.00	
Lung	260	1.89 (1.38–2.59)	<0.001	1.94 (1.34–2.82)	<0.001
Other	340	1.35 (1.01–1.79)	0.045	1.47 (1.02–2.09)	0.037
ECOG					
0–1	572	1.00		1.00	
2	283	4.22 (3.11–5.72)	<0.001	3.68 (2.65–5.11)	<0.001
3–4	97	16.56 (8.42–32.58)	<0.001	13.11 (6.45–26.66)	<0.001
BMI‐adjusted WLGS					
Grade 0	332	1.00			
Grade 1	212	1.14 (0.80–1.62)	0.472	1.16 (0.78–1.73)	0.463
Grade 2	111	1.67 (1.01–2.57)	0.021	1.69 (1.04–2.73)	0.034
Grade 3	181	2.36 (1.63–3.42)	<0.001	2.06 (1.37–3.11)	0.001
Grade 4	94	5.05 (3.00–8.49)	<0.001	4.29 (2.44–7.55)	<0.001

Cases available for analysis: *n* = 886. BMI‐adjusted WLGS, body mass index‐adjusted weight loss grading system; CI, confidence interval; ECOG, Eastern Cooperative Oncology Group; OR, odds ratio.

a Multivariate model adjusted for sex, age, cancer site, and ECOG PS.

## Discussion

Our findings highlight that the WLGS is capable of identifying patients at risk of poorer QoL. In particular, WL grade 4 is associated with a particularly poor prognosis and increased symptom burden.

We report significant deteriorations in both EORTC QLQ‐C30 functional and symptom scores with increasing WL grades. In terms of symptom burden, appetite loss, pain, dyspnoea, insomnia, and fatigue increased by a median score of >20 points between WL grade 0 and WL grade 4, which is considered to be a large clinically significant difference. Similar results were observed in physical and role functioning scores across WL grades 0–4 (Δ > 20 points), whereas cognitive, social, and emotional functioning deteriorated by between 10 and 20 points, indicating a moderate clinical difference. Importantly, the overall QoL summary score decreased across WL grades from 83.2 in WL grade 0 to 62.7 in WL grade 4 (*P* < 0.001), and on multivariate analysis, higher grades of WL were independently associated with poorer overall summary QoL compared with WL grade 0. Our findings reflect those reported by Vagnildhaug and colleagues, whereby all cachexia domains (dietary intake, performance score, appetite, and fatigue) significantly decreased with increasing WL grades.^8^

The WLGS was the first step towards data‐driven approaches for the development of robust diagnostic criteria for cancer cachexia that relate optimally to meaningful patient‐centred outcomes (e.g. survival). However, whether the WLGS alone is sufficient to identify cancer cachexia or its stages is unknown. Blum *et al*. reported that WL and BMI can distinguish between cachectic and non‐cachectic patients, but neither WL nor BMI is sufficient to classify patients into more than two stages of cachexia.^16^ Cachexia represents a spectrum of conditions and can range in severity and clinical presentation from pre‐cachexia, identified by early clinical and metabolic signs, to refractory cachexia, where extensive muscle and fat depletion is evident, and patients are often immune‐compromised.^1^ Recognition of these stages of cachexia is important as these stages have different implications in the anabolic therapy response. Vagnildhaug *et al*.^8^ reported that the WLGS was predictive of the likelihood of cachexia progression, such that progression to more severe WL grades was greater in patients with WL grade 2 (39%) compared with WL grades 0 (19%) and 1 (22%). Supporting this observation was that patients with WL grade 2, similar to our findings, had a higher cachexia‐related symptom load and poorer physical function compared with lower WL grades; thus, the authors suggested that WL grade 2 was fitting with the ‘pre‐cachexia’ phase. Identification of the pre‐cachexia stage is of great clinical importance as this is the stage in which cachexia treatment should be initiated to achieve maximal response. Although the relationship between the WLGS and stages of cachexia^5^ is not established and is outside the remit of the present study, it would be reasonable to assume that patients with WL grade 4 have refractory cachexia. This is the phase in which patients experience depletion of fat reserves, severe muscle wasting, and immunosuppression, and nutritional treatment initiated in this phase often fails to show clinical benefits.^5^ In the present findings, supporting this observation, WL grade 4 was associated with significantly shorter survival [HR 1.87 (95% CI: 1.42–2.45), *P* < 0.001] and reduced QoL in numerous functional and symptom domains.

In addition to the ability of the WLGS in detecting poor prognosis and QoL, it remains to be determined if the WLGS is capable of identifying patients at risk of treatment and post‐operative complications who may benefit from pre‐treatment rehabilitation. Recently, the WLGS was identified as an independent risk factor for post‐operative complications in a cohort of 84 patients undergoing colorectal cancer resection. Patients with a WL grade ≥3 were at almost double the risk of experiencing a grade II or higher complication post‐surgery [risk ratio (RR) 1.90 (95% CI: 1.22–3.39), *P* = 0.048].^17^

Patients identified with high grade WL who are at risk of poorer QoL may benefit from nutritional support aimed at attenuating WL. Nutritional support can be provided orally through dietary counselling ideally by a registered dietitian, incorporating energy and protein‐dense diets, food fortification, and oral nutritional supplements or a combination of all three.^4^ Importantly, nutritional interventions have proven successful in improving some aspects of QoL in patients with cancer. In a recent systematic review and meta‐analysis examining oral nutritional interventions in malnourished patients with cancer, Baldwin *et al*. reported significantly greater benefits to emotional functioning, dyspnoea, loss of appetite, and global QoL in patients receiving oral nutritional interventions compared with routine care. Importantly, the improvements in QoL were consistent with both small and large differences in scores and likely to be clinically meaningful.^18^, ^19^ ESPEN guidelines on nutrition support in oncology now outline Grade A evidence to support the use of intensive dietary advice with or without the use of oral nutritional supplements to increase dietary intakes and prevent WL during radiotherapy and chemotherapy.^7^ Nutritional support may be best suited towards patients with WL grade 1 or 2, which also have impaired QoL, whereas patients with high grade WL (e.g. 3 or 4) and advanced disease may benefit from early referral to palliative care services, irrespective of formal assessment of symptom burden, as the findings from the present study demonstrate that these patients have markedly impaired QoL and survival.

Our study is associated with a number of strengths and limitations. Although our cohort is composed of a relatively large sample of patients with incurable disease, the tumour group is heterogeneous. Our data were limited in that patients typically had a good PS and were early in their disease trajectory; therefore, these findings may not be representative of the entire population. In addition, our findings cannot be extrapolated to other populations (e.g. cancer survivors), and therefore, further research is required to validate these findings in external cohorts of patients. It is also challenging to disentangle if differences in QoL across WL categories simply reflect more advanced disease, increased disease burden, and resultant increased symptom burden or are directly related to WL. Further, the findings of the present study do not assess whether the WLGS is superior to other measures associated with reduced survival such as ECOG PS, the latter having a clear relationship with QoL and widely used clinically. However, the present findings suggest that WL grades were associated with poorer QoL scores independent of ECOG PS, highlighting that both have an impact on summary QoL score. Future work that examines these in combination would be of interest. Data on WL prior to 3 months were not available for the cohort described herein. This may have been interesting to ascertain the recent degree of WL compared with overall WL. Importantly, longitudinal measures of body composition were not examined in this analysis, and therefore, the composition of weight lost was unknown (skeletal muscle vs. adipose tissue). Given the convenience recruitment strategy, patients may have been at different time points of their disease trajectory when QoL was assessed, which may have influenced QoL scores. In addition, we did not document if patients received oral nutritional supplements and enteral or parenteral nutrition or if they were prescribed any medications that might influence appetite and weight gain.

We report that the WLGS may be useful in identifying patients at risk of poor QoL. WL grade 4 is independently associated with a particularly poor prognosis and increased symptom burden, and identification of patients with WL grade 4 could be useful in identifying those who may benefit from early referral to palliative care services.

## Conflict of interest

None declared.

## Funding

This publication has emanated from research conducted with the financial support of Science Foundation Ireland (SFI) under grant number SFI/12/RC/2273 and Medical Research Scotland (487 FRG).
